# Peripheral endocannabinoids in eating disorders and obesity and its relationship with clinical and anthropometric variables

**DOI:** 10.1192/j.eurpsy.2021.329

**Published:** 2021-08-13

**Authors:** I. Baenas-Soto, R. Miranda-Olivos, L. Vos, R. Granero, I. Sánchez, N. Riesco, A. Del Pino-Gutiérrez, E. Codina, J. A. Fernández-Formoso, N. Vilarrasa, N. Virgili, R. Lopez-Urdiales, A. Pastor, R. De La Torrre, S. Jimenez-Murcia, C. Soriano-Mas, F. Fernandez-Aranda

**Affiliations:** 1 Psychiatry, Bellvitge Hospital - IDIBELL, Barcelona, Spain; 2 Medicina, Universidad Barcelona, Barcelona, Spain; 3 Department Of Psychobiology And Methodology, Autonomous University of Barcelona, Barcelona, Spain; 4 Ciber Fisiopatología Obesidad Y Nutrición (ciberobn), Instituto de Salud Carlos III, Madrid, Spain; 5 Department Of Public Health, Mental Health And Perinatal Nursing, School Of Nursing, University of Barcelona, Barcelona, Spain; 6 Ciberdem-ciber De Diabetes Y Enfermedades Metabólicas Asociadas, Instituto de Salud Carlos III, Madrid, Spain; 7 Department Of Endocrinology And Nutrition, University Hospital of Bellvitge-IDIBELL, Barcelona, Spain; 8 Integrative Pharmacology And Systems Neuroscience Research Group, Hospital del Mar Research Institute (IMIM), Barcelona, Spain; 9 Department Of Experimental And Health Sciences, Universitat Pompeu Fabra (CEXS-UPF), Barcelona, Spain; 10 Psychiatry, University Hospital Bellvitge-IDIBELL and CIBEROBN, Barcelona, Spain; 11 Department Of Psychiatry, University Hospital of Bellvitge-IDIBELL, CIBERobn, University of Barcelona, Barcelona, Spain; 12 Psychiatry, Bellvitge University Hospital-IDIBELL, Barcelona, Spain; 13 Psychiatry, University Hospital Bellvitge-IDIBELL and CIBERobn, Barcelona, Spain

**Keywords:** Anandamide, eating disorders, obesity, 2-Arachidonoylglycerol

## Abstract

**Introduction:**

Anandamide (AEA) and 2-Arachidonoylglycerol (2-AG) play a pivotal role in food intake and reward aspects of feeding. Aberrant functioning in the endocannabinoid system has been observed in patients with eating disorders (EDs). This dysfunction may influence the incentive processes stimulating behaviors towards food acquisition or the hedonic evaluation of ingested food.

**Objectives:**

The aims of this study are to compare fasting peripheral levels of AEA and 2-AG in ED patients, obese subjects (OB) and healthy controls (HCs), and to explore their association with clinical and anthropometric variables.

**Methods:**

The sample included a total of 63 adult women. Peripheral blood samples were collected to investigate fasting levels of AEA and 2-AG in 31 ED patients: 22 Anorexia Nervosa (AN) and 9 Binge Eating Disorder (BED), compared to 21 OB and 11 HCs. Several clinical and anthropometric variables were also assessed.

**Results:**

Comparing groups, significant differences in AEA levels were found (p=0.001). Specifically, individuals with AN exhibited lower AEA than OB (p<0.001) and BED (p=0.007), while OB showed higher AEA than HCs (p=0.015). 2-AG was positively correlated with hostility dimension in EDs and negatively associated with impulsive traits in OB. AEA showed a direct association with body dissatisfaction in AN, contrary to OB. Finally, in AN, AEA negatively correlated with the body mass index, while 2-AG was positively associated with the fat mass.

**Conclusions:**

These results suggest an interaction between biological and clinical factors defining a vulnerability pathway that could help fitting personalized therapeutic approaches in each condition.

**Disclosure:**

No significant relationships.

